# A combined model integrating deep learning, radiomics, and clinical ultrasound features for predicting *BRAF V600E* mutation in papillary thyroid carcinoma with Hashimoto’s thyroiditis

**DOI:** 10.3389/fendo.2025.1641037

**Published:** 2025-08-18

**Authors:** Peng-Fei Zhu, Xiao-Feng Zhang, Pu Zhou, Jiang-Yuan Ben, Hao Wang, Shu-E Zeng, Xin-Wu Cui, Ying He

**Affiliations:** ^1^ Department of Ultrasonic Medicine, Nantong Tumor Hospital, Nantong, Jiangsu, China; ^2^ Department of Medical Ultrasound, Tongji Hospital, Tongji Medical College, Huazhong University of Science and Technology, Wuhan, China; ^3^ Department of Ultrasound, The First Affiliated Hospital of Xinxiang Medical University, Xinxiang, Henan, China; ^4^ Department of Ultrasound, Hubei Cancer Hospital, Wuhan, China

**Keywords:** papillary thyroid carcinoma, Hashimoto’s thyroiditis, BRAF V600E mutation, radiomics, deep learning, ultrasound

## Abstract

**Objective:**

This study aims to develop an integrated model that combines radiomics, deep learning features, and clinical and ultrasound characteristics for predicting *BRAF V600E* mutations in patients with papillary thyroid carcinoma (PTC) combined with Hashimoto’s thyroiditis (HT).

**Methods:**

This retrospective study included 717 thyroid nodules from 672 patients with PTC combined with HT from four hospitals in China. Deep learning and radiomics were employed to extract deep learning and radiomics features from ultrasound images. Feature selection was performed using Pearson’s correlation coefficient, the Minimum Redundancy Maximum Relevance (mRMR) algorithm, and LASSO regression. The optimal algorithm was selected from nine machine learning algorithms for model construction, including the traditional radiomics model (RAD), the deep learning model (DL), and their fusion model (DL_RAD). Additionally, a final combined model was developed by integrating the DL_RAD model with clinical and ultrasound features. Model performance was assessed using AUC, calibration curves, and decision curve analysis (DCA), while SHAP analysis was used to interpret the contribution of each feature to the combined model’s output.

**Results:**

The combined model achieved superior diagnostic performance, with AUC values of 0.895, 0.864, and 0.815 in the training, validation, and external test sets, respectively, outperforming the RAD model, DL model, and RAD_DL model. DeLong test results indicated significant differences in the external test set (*p<*0.05). Further validation through calibration curves and DCA confirmed the model’s robust performance. SHAP analysis revealed that RAD_DL signature, aspect ratio, extrathyroidal extension, and gender were key contributors to the model’s predictions.

**Conclusion:**

The combined model integrating radiomics, deep learning features, and clinical as well as ultrasound characteristics exhibits excellent diagnostic performance in predicting *BRAF V600E* mutations in patients with PTC coexisting with HT, highlighting its strong potential for clinical application.

## Introduction

Thyroid cancer is the most common endocrine malignancy, with its incidence steadily increasing in recent years, making it a global health concern (1, 2). Papillary thyroid carcinoma (PTC) is the predominant subtype, accounting for approximately 80% of all thyroid cancers (3). Although PTC typically follows an indolent course with a favorable prognosis, accumulating evidence has revealed substantial heterogeneity in its biological behavior (4). While some cases can be safely managed through active surveillance, thereby avoiding surgical complications such as permanent hypoparathyroidism and vocal cord injury, others exhibit aggressive features, including lymph node metastasis(LNM), local recurrence, and even distant spread (5, 6).

Among the molecular alterations identified, the *BRAF V600E* mutation has emerged as a key driver underlying these divergent clinical outcomes. It is the most common genetic alteration in PTC, with a reported mutation frequency of 40% to 80% (7, 8). This mutation leads to the constitutive activation of the MAPK signaling pathway, promoting tumor cell proliferation, differentiation, and invasion (9, 10). Previous studies have demonstrated that PTC patients harboring the *BRAF V600E* mutation are more prone to extrathyroidal extension (ETE), LNM, and local recurrence, while also exhibiting reduced sensitivity to radioactive iodine (RAI) therapy, ultimately affecting long-term prognosis (11–13). Consequently, the *BRAF V600E* mutation is recognized as a crucial biomarker of PTC aggressiveness, playing a significant role in guiding surgical strategies, RAI treatment planning, and follow-up management (14).

Currently, ultrasound-guided fine-needle aspiration (FNA) combined with genetic testing is the primary clinical method for detecting *BRAF V600E* mutations (15). Although FNA has high diagnostic value, it is associated with certain limitations, including its invasive nature, potential complications (such as bleeding and infection), poor patient compliance, and the need for operators with advanced technical expertise, which restricts its widespread adoption in primary healthcare settings (16–18). Therefore, exploring non-invasive and efficient methods for predicting *BRAF V600E* mutations is of great clinical significance for achieving precision treatment in PTC.

In recent years, radiomics has enabled the high-throughput extraction of quantitative imaging features, providing deeper biological insights into tumors beyond conventional imaging techniques (19). It has been widely used to predict tumor malignancy, molecular characteristics, and other pathological features. However, since radiomics relies on manually defined features, it may limit the extraction of deeper imaging features. Meanwhile, deep learning, particularly convolutional neural networks (CNNs), has achieved groundbreaking advancements in medical image analysis (20). By leveraging multilayer neural network architectures, CNNs can autonomously learn and extract high-dimensional, nonlinear features, enabling the identification of microscopic structures that are challenging to detect using traditional imaging analysis (21). Despite the superior feature extraction capabilities of deep learning, its interpretability remains limited, and it often overlooks the potential value of clinical information. This limitation may hinder the clinical applicability and generalizability of the models.

Furthermore, Hashimoto’s thyroiditis (HT) is the most common comorbidity associated with PTC and has been increasingly prevalent in recent years (22). Its chronic inflammatory microenvironment may have a profound impact on the biological behavior and molecular characteristics of PTC, including the regulation of *BRAF V600E* mutation status (23, 24). Studies have reported significant differences in tumor characteristics, invasive potential, and immune landscape between PTC patients with coexisting HT and those with isolated PTC (25, 26). However, current research on predicting *BRAF V600E* mutations in PTC patients with HT remains limited, partly because the inflammatory microenvironment in HT may alter the mutation’s role in tumor progression (27). Therefore, developing a prediction model specifically for PTC patients with HT is essential to clarify the role of *BRAF V600E* mutations in this subgroup and to facilitate more personalized therapeutic strategies.

Given the critical influence of the inflammatory microenvironment in HT on the progression of PTC harboring *BRAF V600E* mutations—and the current lack of research specifically addressing the prediction of such mutations in this unique subset—this study integrates radiomics, deep learning, clinical, and ultrasound features to construct an ultrasound-based prediction model tailored for PTC with HT. This model is designed to offer a non-invasive, accurate, and generalizable method for the preoperative prediction of *BRAF V600E* mutations. By enabling precise risk stratification, it may support personalized management strategies for PTC patients with HT, enhancing the identification of high-risk individuals while potentially avoiding overtreatment in low-risk cases.

## Methods

### Study population

**Figure 1:**
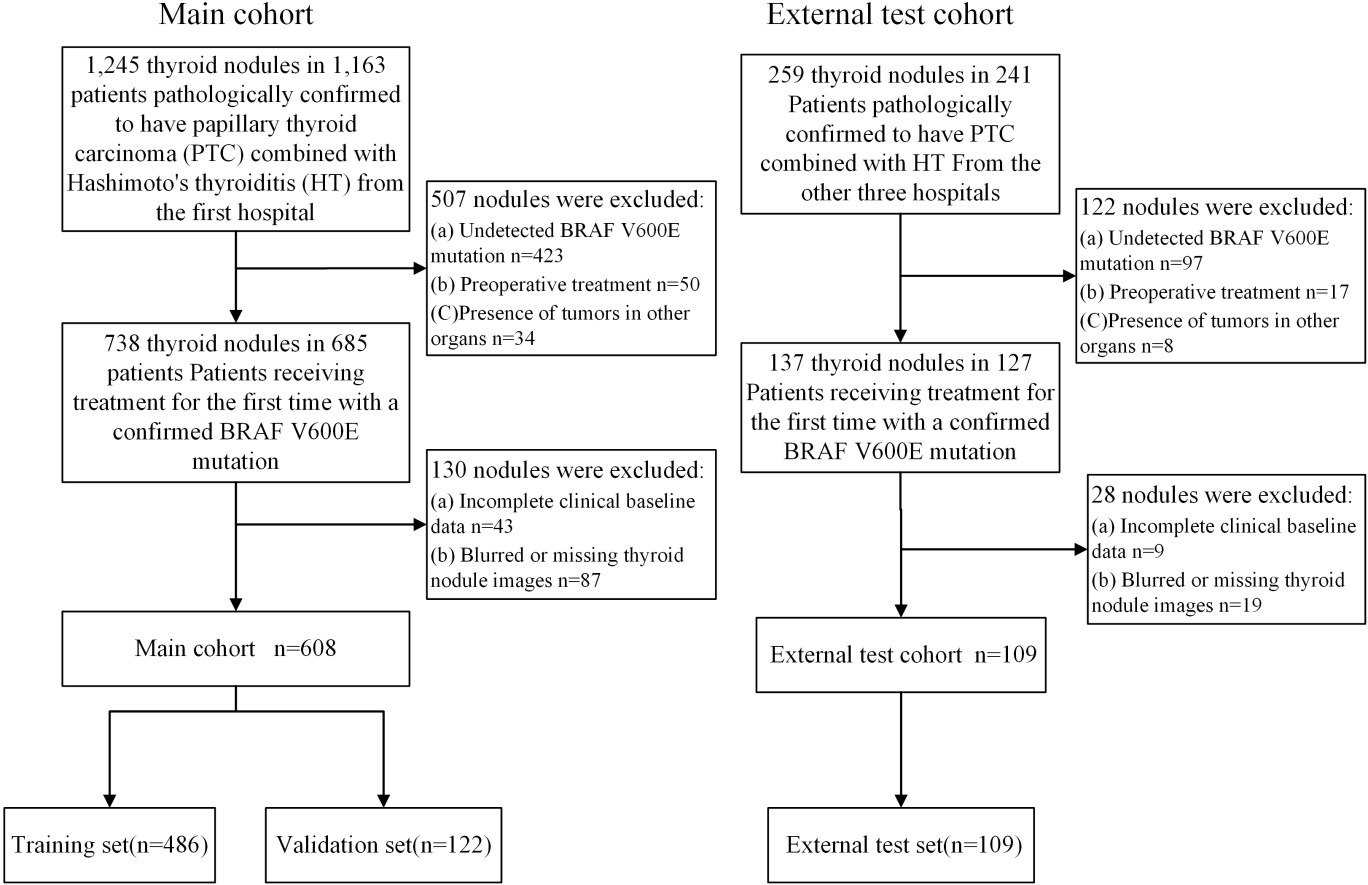
The inclusion process of the study population.

This retrospective study was approved by the Institutional Review Board (No.2024-A 06). A total of 717 nodules from 672 patients with PTC combined with HT were collected from four hospitals in China. The training and validation sets consisted of 608 nodules from 570 patients who underwent surgery at the Tongji Hospital of Huazhong University of Science and Technology, from June 12, 2017, to March 21, 2024. Since each thyroid nodule exhibits distinct imaging and pathological characteristics, we treated each nodule as an independent unit. The training and validation sets were then randomly divided in an 8:2 ratio at the nodule level. The external test set included 109 nodules from 102 patients from Hubei Cancer Hospital, Nantong First People’s Hospital, and the First Affiliated Hospital of Xinxiang Medical University, between March 21, 2022, and November 7, 2024 (**Figure 1**).

Inclusion Criteria: 1. Pathologically confirmed diagnosis of PTC with HT. 2. Clear *BRAF V600E* mutation status. 3. First-time thyroid surgery. 4. Ultrasound examination performed within two weeks before surgery. 5. Availability of complete and clear thyroid nodule images. 6. Complete clinical data. Exclusion Criteria: 1. Unclear *BRAF V600E* mutation status. 2. Blurred or missing thyroid nodule images. 3. Missing baseline clinical data. 4. Previous treatment (e.g., thyroid ablation or surgery) prior to the current surgery. 5. Presence of tumors in other organs.

### Clinical data collection

The clinical data collected included the patient’s gender, age, and pathological information, including *BRAF V600E* mutation status. The ultrasound feature of aspect ratio was also recorded and dichotomized as >1 vs. ≤1. This threshold was adopted based on the ACR TI-RADS guideline, where an aspect ratio >1 (taller-than-wide) is considered suggestive of malignancy and is widely used in clinical thyroid risk stratification systems.

### Ultrasound image acquisition

The ultrasound devices used in this study include LOGIQ E20 (GE Healthcare, Wauwatosa, USA), Affiniti 70 (Philips Healthcare, Suzhou, China), DD70 (DDIT, Shenzhen, China), LOGIQ E9 (GE Healthcare, Wauwatosa, USA), EPIQ 5 (Philips Healthcare, Andover, USA), EPIQ 7 (Philips Healthcare, Andover, USA), LOGIQ S8 (GE Healthcare, Wauwatosa, USA), Resona 9S (Mindray, Shenzhen, China), and RS85 (Samsung Medison, Seoul, South Korea).

During the examination, the patient was positioned supine with the head slightly tilted backward to fully expose the neck region. All ultrasound examinations were performed by experienced sonographers with over five years of clinical experience, following a standardized scanning protocol. The sonographer selected the largest cross-sectional view of the thyroid nodule and captured high-quality images. Detailed information on nodule size, location, aspect ratio, shape, internal echogenicity, calcification, and ETE was carefully recorded.

### Data preprocessing and region of interest delineation

Tumor regions were delineated by physicians with over five years of experience, without prior knowledge of the *BRAF V600E* mutation status. To assess the consistency of radiomics features, 100 thyroid nodules were randomly selected, and two radiologists, each with more than five years of experience, independently delineated the tumor regions without knowing the *BRAF V600E* mutation status. Inter-observer consistency was evaluated using the intraclass correlation coefficient (ICC), with an ICC value ≥0.75 indicating good consistency. Features with an ICC below this threshold were excluded to ensure the stability and reproducibility of the radiomic features.

### Radiomics feature extraction

Radiomic features from the ROI were extracted using Pyradiomics (https://pyradiomics.readthedocs.io/en/latest/index.html), including first-order features such as mean, standard deviation, kurtosis, and skewness; shape features such as volume, aspect ratio, and boundary irregularity; texture features including the Gray Level Co-occurrence Matrix (GLCM), Gray Level Run Length Matrix (GLRLM), Gray Level Size Zone Matrix (GLSZM), and Nearest-Neighbor Gray Tone Difference Matrix (NGTDM); and wavelet features. A total of 1208 radiomics features were extracted.

### Deep learning feature extraction

In this study, ResNet18 was used to extract deep learning features from ultrasound images. First, the largest rectangular bounding box of ROI was cropped, and all images were uniformly resized to 224×224 pixels to ensure consistency in input scale. To enhance the model’s generalization ability, various data augmentation strategies were applied, including random horizontal flipping, random brightness adjustment, and random rotation. The model was initialized with ImageNet pre-trained weights to accelerate convergence and improve feature extraction capability. During training, the Stochastic Gradient Descent (SGD) optimizer was used, with an initial learning rate of 0.01, 50 total epochs, and a cross-entropy loss function. Deep learning features were extracted from the output of the final global average pooling (AvgPool) layer of ResNet18, yielding a 512-dimensional deep learning feature vector for subsequent model analysis.

### Feature selection

To reduce feature redundancy and optimize model performance, Spearman correlation analysis was first employed to assess the correlation between features. For features with a correlation coefficient greater than 0.9, only the one with higher information value was retained. Additionally, the Minimum Redundancy Maximum Relevance (mRMR) algorithm was applied (28). Subsequently, feature selection was performed using Least Absolute Shrinkage and Selection Operator(LASSO) regression (29). LASSO applied L1 regularization to shrink some of the regression coefficients to zero, thereby eliminating irrelevant features. The remaining non-zero coefficient features were used to construct the machine learning model. Details of the complete feature selection workflow and results are provided in **Supplementary File 1**.

### Model construction

This study developed three predictive models based on radiomics (Rad Model), deep learning (DL Model), and the fusion of both (DL_RAD Model). Additionally, a comprehensive model combining radiomics, deep learning, and clinical and ultrasound features was constructed (Combined Model). Each model was compared using nine machine learning algorithms: Logistic Regression (LR), Support Vector Machine (SVM), Random Forest (RF), ExtraTrees (ET), K-Nearest Neighbors (KNN), XGBoost (XGB), LightGBM (LGBM), Gradient Boosting (GB), and Multilayer Perceptron (MLP), with the best-performing model selected. For different feature inputs, all training set data were randomly split in a 7:3 ratio, with 70% used for training and 30% for testing. Hyperparameter optimization was performed using 5-fold cross-validation to improve model stability and generalization capability.

**Figure 2:**
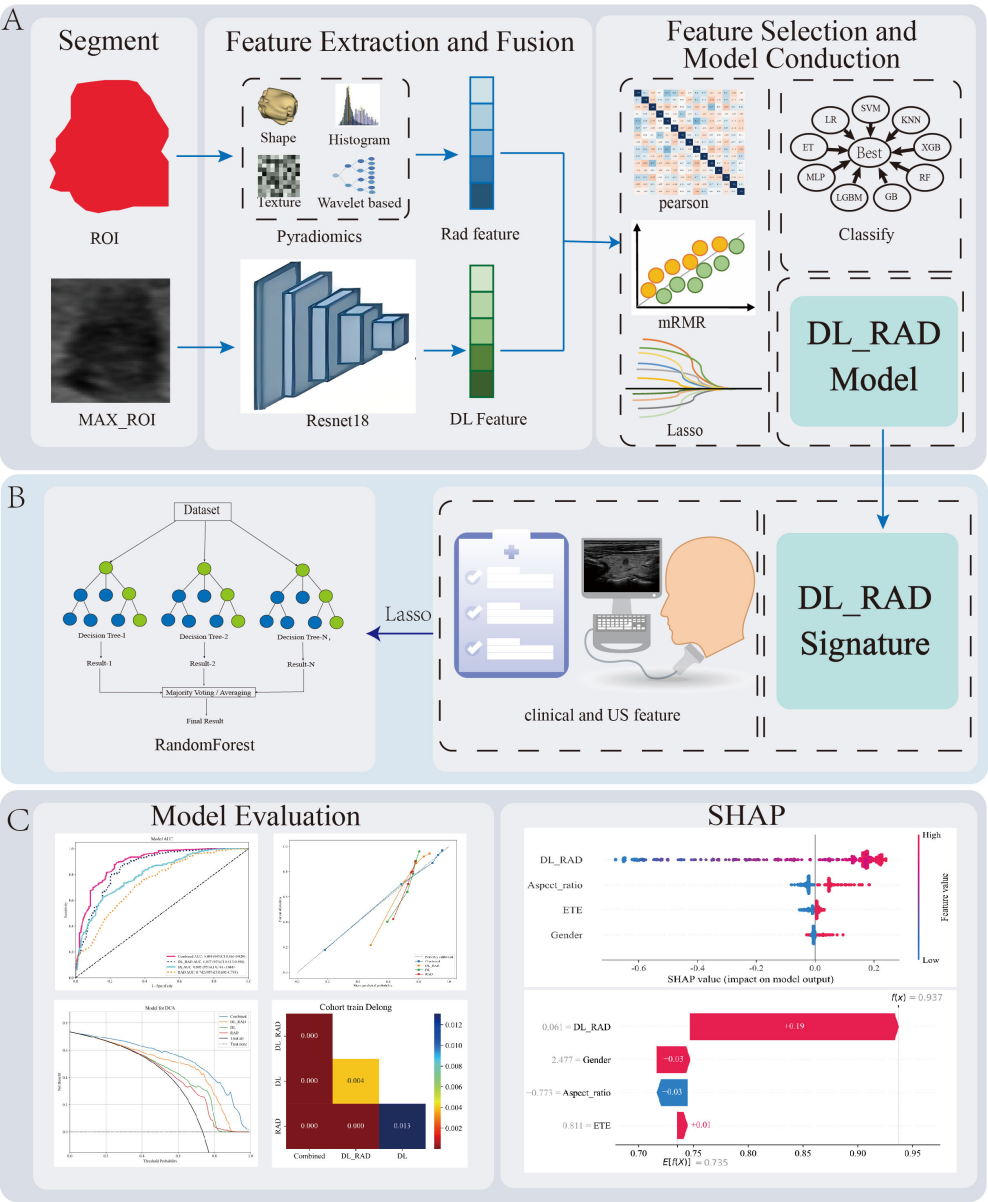
Workflow diagram for the development and evaluation of predictive models. **(A)** Image preprocessing, radiomics and deep learning feature extraction, feature selection, feature fusion, and construction of the DL_RAD model. **(B)** Construction of the Combined model by integrating the DL_RAD signature with clinical and ultrasound features. Additionally, among the nine machine learning algorithms, random forest achieved the best performance. **(C)** Model evaluation and interpretation. LR, Logistic Regression; SVM, Support Vector Machine; RF, Random Forest; XGB, XGBoost; KNN, K-Nearest Neighbors; LGBM, LightGBM; ET, ExtraTrees; G, Gradient Boosting; MLP, Multilayer Perceptron.

Rad Model uses the selected radiomics features as input, while the DL Model is built using the selected ResNet18 deep learning features. The DL_RAD Model adopts early feature fusion, combining radiomics features and deep learning features before feature selection, and the selected features are then used for training to construct the DL_RAD model. The Combined Model integrates clinical parameters, ultrasound features, and the DL_RAD model signature, and after feature selection, the same nine machine learning algorithms are applied to obtain the optimal model. **Figure 2**. Workflow and technical roadmap for the development and evaluation of predictive models.

### Model evaluation and interpretability

The classification performance of the models was quantified using the Receiver Operating Characteristic (ROC) curve and Area Under the Curve (AUC) to determine the best predictive model. Decision Curve Analysis (DCA) was applied to assess the clinical net benefit of the models at different decision thresholds, helping to evaluate their practical application value. Model calibration was assessed using a calibration curve to analyze the consistency between predicted probabilities and actual incidence rates. To improve the interpretability of the models, SHapley Additive exPlanations (SHAP) was used to explain the Combined Model, quantifying the contribution of each feature to the model’s predictions and revealing its decision logic (30). The SHAP method, based on Shapley values, quantifies the contribution of each feature to the model’s predictions and reveals the decision logic both globally and at the individual level. Global SHAP analysis provides an importance ranking of different features, while individual SHAP analysis visually displays the driving factors of predictions for each sample, enhancing the model’s transparency and clinical interpretability.

### Statistical analysis

Statistical analysis of patient baseline data was performed using R software (version 4.3.3, https://www.r-project.org) and the compareGroups package. Continuous variables were summarized as mean ± standard deviation, while categorical variables were presented as frequencies and percentages. The normality of continuous variables was assessed using the Shapiro-Wilk test. For continuous variables that did not follow a normal distribution, data were presented as median with interquartile ranges (IQRs), and group comparisons were performed using the non-parametric Mann–Whitney U test. For group comparisons, continuous variables were evaluated using the Mann-Whitney U test or Student’s t-test, while categorical variables were assessed using the Chi-squared test or Fisher’s exact test. Additionally, the DeLong method was used to compare the area under the curve (AUC) of different models to assess their predictive performance. All statistical analyses were conducted using two-sided tests, with a significance threshold of p < 0.05.

## Results

### Patient characteristics

This retrospective study included a total of 717 nodules (age 41.97 ± 11.16, 102 males, 615 females), with the training set comprising 486 nodules (age 41.39 ± 10.99, 68 males, 418 females), the validation set comprising 122 nodules (age 42.62 ± 11.77, 17 males, 105 females), and The external test set comprising 109 nodules (age 43.84 ± 11.11, 17 males, 92 females) (**Table 1**). There were no significant differences in clinical and ultrasound features across the three datasets.

**Table 1:** Baseline characteristics of study sets.

	Training	Validation	External test	p.overall
	*N=486*	*N=122*	*N=109*	
Gender:				0.906
Female	418 (86.0%)	105 (86.1%)	92 (84.4%)	
Male	68 (14.0%)	17 (13.9%)	17 (15.6%)	
Age	40.0 [33.0;50.0]	42.5 [33.0;52.8]	43.0 [35.0;52.0]	0.102
Size	0.80 [0.60;1.20]	0.80 [0.60;1.20]	0.70 [0.60;1.10]	0.096
Aspect_ratio:				0.196
≤1	304 (62.6%)	74 (60.7%)	58 (53.2%)	
>1	182 (37.4%)	48 (39.3%)	51 (46.8%)	
Calcification:				0.099
Absent	181 (37.2%)	58 (47.5%)	40 (36.7%)	
Present	305 (62.8%)	64 (52.5%)	69 (63.3%)	
ETE:				0.696
Absent	193 (39.7%)	46 (37.7%)	47 (43.1%)	
Present	293 (60.3%)	76 (62.3%)	62 (56.9%)	
Location:				0.181
Left	231 (47.5%)	50 (41.0%)	59 (54.1%)	
Right	242 (49.8%)	71 (58.2%)	47 (43.1%)	
Isthmus	13 (2.67%)	1 (0.82%)	3 (2.75%)	
Internal Echogenicity:				0.288
Homogeneous	3 (0.62%)	1 (0.82%)	2 (1.83%)	
Heterogeneous	483 (99.4%)	121 (99.2%)	107 (98.2%)	
Shape:				0.582
Regular	15 (3.09%)	5 (4.10%)	5 (4.59%)	
Irregular	471 (96.9%)	117 (95.9%)	104 (95.4%)	
*BRAF V600E*:				0.867
Negative	129 (26.5%)	34 (27.9%)	27 (24.8%)	
Positive	357 (73.5%)	88 (72.1%)	82 (75.2%)	

ETE, extrathyroidal extension.

### The performance of the RAD and DL model

In this study, the RAD and DL models were constructed using radiomics features and deep learning features, respectively. All results are summarized in **Table 2**. Among the nine machine learning algorithms, ExtraTrees performed the best in both models. The RAD model achieved good performance in the training and validation sets, with AUC values of 0.742 (95% CI: 0.692–0.793) and 0.721 (95% CI: 0.613–0.829), respectively. However, its generalizability was limited, as evidenced by a markedly reduced AUC of 0.518 (95% CI: 0.391–0.643) in the external test cohort. Despite a relatively high accuracy of 0.706 in the external test cohort, the F1 score was 0.812, and the Youden’s index was only 0.137. The DL model showed improved predictive performance, with AUC values of 0.805 (95% CI: 0.761–0.847), 0.776 (95% CI: 0.684–0.867), and 0.704 (95% CI: 0.602–0.778) in the training, validation, and external test sets, respectively. Nevertheless, the model remained suboptimal, with values of 0.619, 0.602, and 0.549 across the respective cohorts.

**Table 2:** Performances of the predictive models in three sets.

Model and metric	AUC	95% CI	ACC	SEN	SPE	PPV	NPV
Training set							
RAD	0.742	0.6918 - 0.7928	0.698	0.714	0.651	0.85	0.452
DL	0.805	0.7612 - 0.8479	0.679	0.619	0.845	0.917	0.445
DL_RAD	0.857	0.8154 - 0.8982	0.833	0.871	0.729	0.899	0.671
Combined	0.895	0.8602 - 0.9289	0.85	0.88	0.767	0.913	0.697
Validation set							
RAD	0.721	0.6131 - 0.8295	0.713	0.739	0.647	0.844	0.489
DL	0.776	0.6838 - 0.8673	0.664	0.602	0.824	0.898	0.444
DL_RAD	0.847	0.7681 - 0.9254	0.836	0.886	0.706	0.886	0.706
Combined	0.864	0.7939 - 0.9337	0.828	0.875	0.706	0.885	0.686
External test set							
RAD	0.518	0.3913 - 0.6439	0.706	0.841	0.296	0.784	0.381
DL	0.704	0.6016 - 0.8072	0.606	0.549	0.778	0.882	0.362
DL_RAD	0.773	0.6675 - 0.8782	0.697	0.659	0.815	0.915	0.44
Combined	0.815	0.7154 - 0.9142	0.826	0.866	0.704	0.899	0.633

AUC, area under the receiver operating characteristic curve; ACC, accuracy; SEN, sensitivity; SPE, specificity; PPV, positive predictive value; NPV, negative predictive value.

### The performance of the DL_RAD model

**Figure 3:**
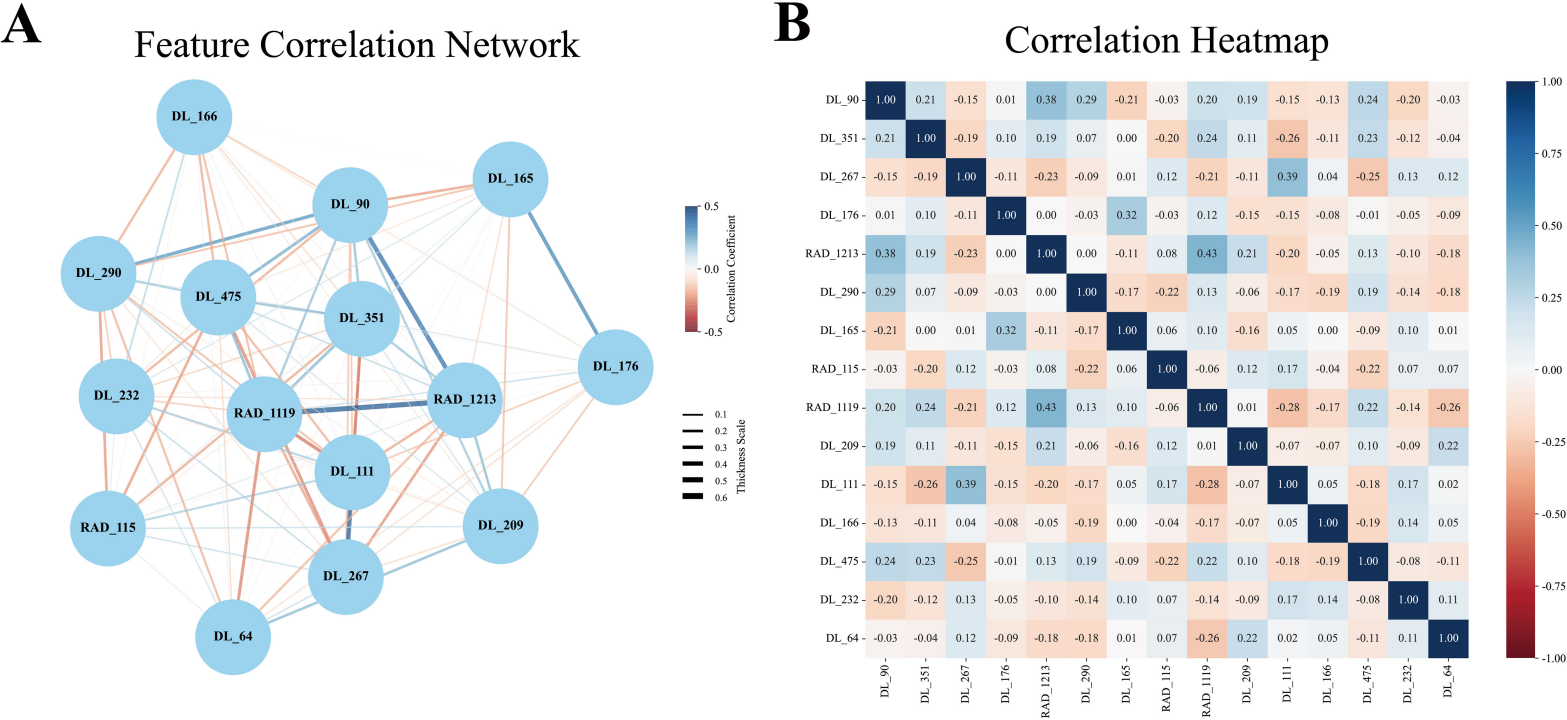
Pearson correlation coefficient network diagram and heatmap. **(A)** The Pearson feature correlation network illustrated the relationships between each pair of selected features. **(B)** The Pearson correlation coefficient heatmap indicated that each feature acted as an independent predictor, as no correlation coefficient exceeded 0.5.

In this study, an early fusion strategy was employed to integrate radiomics and deep learning features, resulting in the construction of a hybrid model (DL_RAD). A total of 15 radiomic and deep learning features were selected to build the DLR model. Network graphs and heatmaps demonstrated relatively low correlations among the features (**Figure 3**). Compared with the individual radiomics and deep learning models, the DL_RAD model demonstrated further improvement in diagnostic performance, with AUC values of 0.857 (95% CI: 0.815–0.898) in the training set, 0.847 (95% CI: 0.768–0.925) in the validation set, and 0.773 (95% CI: 0.667–0.878) in the external test set. While the model exhibited high sensitivity in the training (0.871) and validation (0.886) cohorts, a notable decline in sensitivity was observed in the external test cohort (0.659), indicating potential overfitting and the need for further optimization to improve generalizability.

### The performance of the combined model

**Figure 4:**
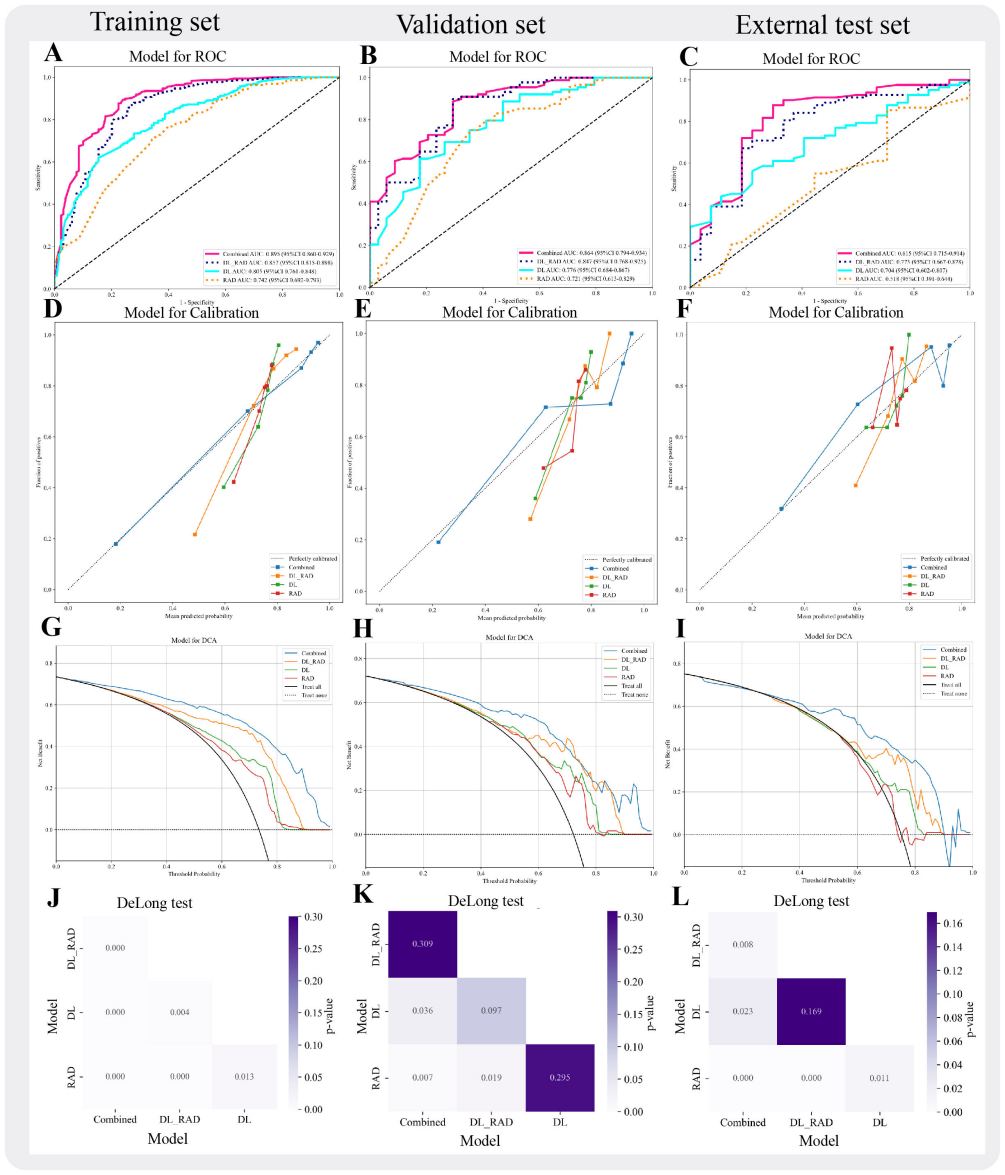
Performance evaluation of different models. **(A-C)** AUC curves for four models (RAD, DL, DL_RAD, Combined) across three sets. **(D-F)** Calibration curves for the four models across the three sets. **(G-I)** Decision curves for the four models across the three sets. **(J-L)** DeLong Test for the four models across the three sets.

Building upon the performance of the DL_RAD model, we further constructed the Combined model by integrating the DL_RAD signature with clinical and ultrasound features, achieving optimal diagnostic performance (**Figures 4A-L**). After feature selection, the final features included Aspect_ratio, ETE, gender, and the DL_RAD signature. Among the nine machine learning algorithms, Random Forest achieved the best classification performance. The AUCs for the Combined model in the training, validation, and external test sets were 0.895 (95% CI: 0.860–0.929), 0.864 (95% CI: 0.794–0.933), and 0.815 (95% CI: 0.715–0.914), respectively. The DeLong test results showed that the AUC of the Combined model in the external test set was significantly higher than that of the other three models (*p <* 0.05) (**Figure 4L**). Additionally, compared to other models, the Combined model demonstrated a significant increase in sensitivity in the external test set, reaching 0.866.

### Model interpretability

**Figure 5:**
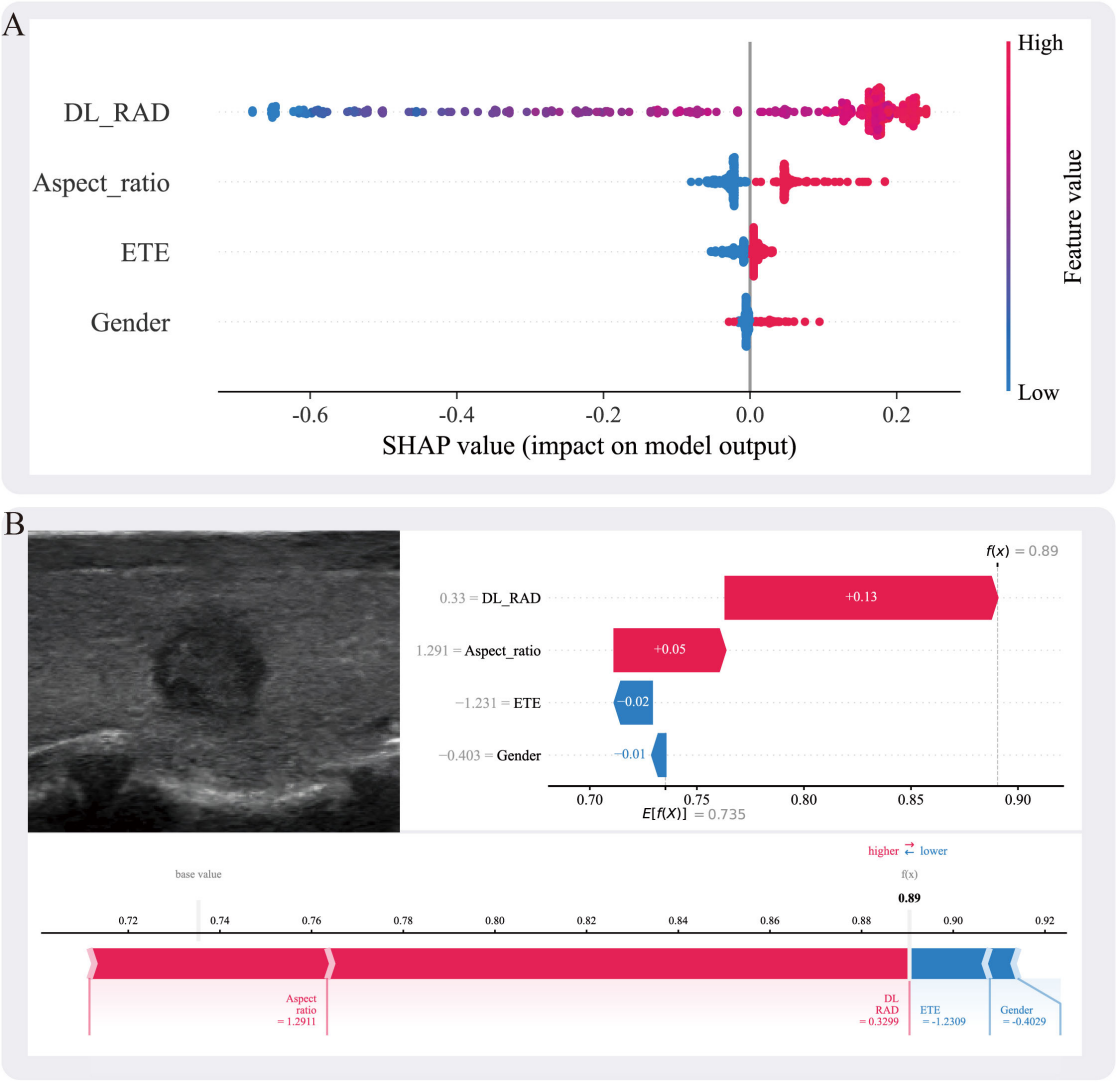
**(A)** The SHAP summary plot illustrates the impact of each feature on the model’s prediction. The features include DL_RAD, aspect ratio, ETE, and gender. Higher SHAP values indicate a greater contribution of the corresponding feature to the prediction outcome. **(B)** Individual prediction explanation for a specific case, where the ultrasound image is shown on the left and the corresponding SHAP force plot on the right. The DL_RAD prediction value, aspect ratio, ETE, and gender are 0.33, 1.291, −1.231, and −0.403, respectively, contributing +0.13, +0.05, −0.02, and −0.01 to the malignant label decision. Among these, the DL_RAD prediction value and aspect ratio positively support the malignant prediction, whereas extrathyroidal extension and gender exert a negative influence. Summing these contributions with the expected value (E[f(X)] = 0.735) yields a final decision probability of 0.89. SHAP values represent absolute contributions to the predicted probability (i.e., additive changes from the base value), measured in probability units.

To enhance the interpretability of the fusion model, this study employed SHapley Additive exPlanations (SHAP) for the explanation analysis of the combined model. The summary plot revealed that Aspect_ratio, extrathyroidal ETE, gender, and the DL_RAD signature all contributed to the Combined model, with the DL_RAD signature having the most significant contribution and gender having the least (**Figure 5A**). **Figure 5B** illustrates the contribution of each feature in the individual case to the Combined model’s prediction of *BRAF V600E* mutation status.

## Discussion

In this study, we developed a Combined model to predict the *BRAF V600E* mutation status in patients with PTC associated with HT, integrating radiomics, deep learning features, as well as clinical and ultrasound characteristics. By comparing nine machine learning algorithms, we significantly enhanced the model’s diagnostic performance. In the training, validation, and test sets, the Combined model achieved optimal performance, with AUC values of 0.895 (95% CI: 0.860–0.929), 0.864 (95% CI: 0.794–0.933), and 0.815 (95% CI: 0.715–0.914), respectively. Furthermore, we used the SHAP method to interpret the model, improving its interpretability and clinical applicability.

*BRAF V600E* mutation is the most common mutation in PTC and has a significant impact on tumor invasiveness, the effectiveness of radioactive iodine therapy, and long-term prognosis (7, 31). Studies have shown that *BRAF V600E*-mutated PTC is more likely to exhibit specific imaging features, such as irregular borders, increased aspect ratio, microcalcifications, and ETE (18, 32). However, certain histological subtypes of PTC may present atypical imaging patterns, which could interfere with the generalizability of image-based predictive models for BRAF V600E mutations (33). To overcome these limitations, the application of artificial intelligence (AI) in medical imaging has advanced significantly in recent years, showing great potential in disease recognition and risk stratification. Deep learning and machine learning techniques have been employed in tasks such as thyroid cancer segmentation, recurrence risk classification, and malignancy prediction, showing promising diagnostic performance (34–36). In addition, successful applications in cross-domain tasks—such as pediatric bone mineral density estimation and abnormal cell detection in FISH images—further support the broad applicability of AI in multimodal medical data analysis (37, 38). Zhang et al. constructed a predictive model for *BRAF V600E* mutation using radiomics based on MRI images (39). This finding further demonstrated the value of imaging features in predicting *BRAF V600E* mutations and suggested that radiomics can quantify microstructural changes in tumors, providing new imaging indicators for the molecular classification of PTC. However, MRI is expensive and time-consuming, which limits its widespread application in clinical practice.

Furthermore, the presence of HT may alter the biological behavior of PTC, making its imaging features and molecular mechanisms significantly different from those of pure PTC (40). Previous studies have indicated that the chronic inflammatory microenvironment characteristic of HT, including the high infiltration of CD8^+^ T cells and sustained activation of the IFN-γ/STAT1 pathway, may enhance immune surveillance and effectively suppress the expansion of mutated clones. It has been reported that the prevalence of BRAF V600E mutations in patients with PTC coexisting with HT is significantly lower than in those without HT, and the activity of the MAPK signaling pathway—such as the expression level of phosphorylated ERK (p-ERK)—is also markedly reduced (27, 41). In addition, HT-associated inflammatory cytokines (such as IFN-γ and TGF-β) may interact with the BRAF-driven MAPK pathway and further influence downstream biological behavior. In the context of HT, reduced MAPK signaling activity may attenuate tumor cell dedifferentiation, stromal remodeling, and epithelial-mesenchymal transition (EMT), which ultimately manifests as more subtle imaging features such as decreased hypoechogenicity, clearer lesion margins, reduced aspect ratio, and fewer microcalcifications (42). These changes may obscure the typical imaging patterns associated with BRAF mutations, thus impairing the model’s ability to accurately identify such mutations. Consequently, direct application of PTC prediction models could lead to reduced predictive performance. Therefore, the development of a specific *BRAF V600E* mutation prediction model for PTC with HT can more precisely capture HT-related imaging and molecular features, enhancing the model’s clinical applicability. In this study, the Combined model achieved AUC values of 0.895, 0.864, and 0.815 in the training, validation, and external test sets, respectively. This result indicates that the Combined model performs well in the complex clinical context of HT, effectively identifying *BRAF V600E* mutations.

This study developed and compared four predictive models: the RAD model, the DL model, the RAD_DL model, and the Combined model to determine the optimal approach for predicting *BRAF V600E* mutation. Radiomics, based on high-throughput imaging feature extraction, quantifies lesion morphology, texture, and statistical properties, providing deeper tumor biological insights beyond traditional imaging (43). In this study, the RAD model achieved AUCs of 0.742 and 0.721 in the training and validation sets, respectively, indicating its ability to identify certain imaging features associated with *BRAF V600E* mutation. However, its AUC dropped to 0.518 in the test set, suggesting poor generalization to new data. This decline may be attributed to handcrafted features failing to fully capture the nonlinear and complex imaging patterns, making the model overly reliant on training data while limiting its adaptability to unseen cases (44). Further analysis revealed a significant class imbalance in the external test set, with *BRAF V600E*-mutated nodules accounting for approximately 75% (82/109) of the cases. This imbalance caused the model to favor the majority class (*BRAF V600E*-positive nodules), achieving a relatively high overall accuracy (0.706) but poor discrimination for negative cases, resulting in a low AUC. Although the RAD model showed high sensitivity (0.841) and a good F1 score (0.812), its specificity was low (0.296), and the Youden’s index was only 0.137, indicating limited overall discriminatory power. These results are consistent with the low AUC and reflect the model’s difficulty in identifying *BRAF V600E*-negative nodules.

Compared to radiomics, deep learning models automatically learn high-dimensional, nonlinear imaging features, particularly excelling in fine-grained feature extraction and pattern recognition (20, 45). In this study, the DL model achieved AUCs of 0.805, 0.776, and 0.704 in the training, validation, and external test sets, respectively, outperforming the RAD model. This suggests that deep learning is more effective in capturing imaging patterns associated with *BRAF V600E* mutation. While the DL model showed low sensitivity in the training (0.619), validation (0.602), and external test sets (0.549), indicating limitations in detecting positive cases. This may be due to potential overfitting, which limits the DL model’s generalization ability.

To leverage the strengths of both approaches, we adopted an early fusion strategy by integrating radiomic and deep learning features into a combined model. The RAD_DL model achieved AUCs of 0.857, 0.847, and 0.773 in the training, validation, and test sets, respectively, significantly outperforming the RAD model and DL model. This improvement may arise from the complementary nature of radiomics, which provides global structural information, and deep learning, which excels at capturing intricate patterns (46). Together, they enable the model to more accurately identify imaging features associated with *BRAF V600E* mutations. Further analysis of feature correlations revealed low interdependence between radiomics and deep learning features in the RAD_DL model, underscoring their complementary roles. By integrating radiomics’ strength in global structural recognition with deep learning’s capacity for detailed pattern extraction, the RAD_DL model achieved enhanced predictive performance and improved generalization ability.

To further enhance model performance, we integrated key clinical and ultrasound features into the DL_RAD model, constructing the Combined model. This model achieved AUCs of 0.895 in the training set, 0.864 in the validation set, and 0.815 in the external test set, demonstrating superior performance compared to all other models. To validate its robustness, DeLong tests were performed, showing no significant difference in the internal validation set but a significant improvement in the external test set, underscoring the superior generalizability of the Combined model over the DL_RAD model. These findings suggest that incorporating clinical and US features allows the model to not only capture imaging characteristics but also leverage patient-specific clinical and ultrasound data, thereby improving predictive efficacy and clinical applicability. In the external test set, the Combined model demonstrated favorable overall performance (AUC = 0.815; accuracy = 0.780). However, its sensitivity (0.866) was notably higher than its specificity (0.704), suggesting that the model tends to favor the identification of *BRAF V600E*-positive nodules under the current default threshold. To address this performance imbalance, threshold adjustment may be considered to tailor the model’s behavior to different clinical scenarios. In practical applications, such as high-risk population screening or preoperative assessment for targeted therapy, missing a *BRAF V600E* -positive case could delay optimal treatment. Therefore, lowering the classification threshold to increase sensitivity is recommended in these settings, maximizing the detection of potential mutation carriers. Conversely, in postoperative follow-up or low-risk patient management, minimizing false positives becomes more critical. In such cases, increasing the threshold to improve specificity can reduce unnecessary psychological burden or overtreatment. To support this adaptive strategy, this study incorporated DCA to evaluate the net clinical benefit of each model across varying risk thresholds, thereby validating their applicability and practical value across diverse clinical contexts.

SHAP analysis quantifies the contribution of each feature to the model’s prediction of *BRAF V600E* mutations, revealing both positive and negative impacts of different features, thereby enhancing the model’s interpretability (30, 47). In this study, global SHAP results identified the RAD_DL signature, aspect ratio, ETE, and gender as key factors in predicting *BRAF V600E* mutations. Previous studies have shown that *BRAF V600E*-mutated thyroid cancers exhibit more aggressive behavior, and aspect ratio and ETE, as critical imaging features of PTC aggressiveness, are often closely associated with *BRAF V600E* mutations (48, 49). Additionally, while the incidence of PTC is significantly higher in females than in males (approximately 3:1), the difference in *BRAF V600E* mutation rates between genders is not significant. Although male PTC patients tend to have more aggressive disease and poorer prognosis, the occurrence of *BRAF V600E* mutations does not differ significantly between genders, resulting in a relatively minor impact of this feature in the SHAP analysis (50).

This study has several limitations: 1. It is a retrospective study, and further prospective validation is needed to enhance the model’s generalization ability and clinical applicability. 2. Although the SHAP method can explain the clinical and ultrasound features in the model, the interpretation of deep learning remains limited. Future work should further explore the interpretability of deep learning models. In addition, the current model is constructed based on static two-dimensional images. In the future, as ultrasound video or three-dimensional volumetric data become available, we plan to further explore 3D CNNs or sequence-based modeling architectures to better capture the spatial and temporal characteristics of ultrasound imaging.

## Conclusion

In this study, a combined model was developed by integrating radiomics and deep learning features with clinical and ultrasound characteristics to predict *BRAF V600E* mutations in patients with PTC coexisting with HT. Compared to other models, the Combined model demonstrated the best performance, showcasing its significant potential for clinical application and providing reliable support for preoperative prediction of *BRAF V600E* mutations. Additionally, the use of the SHAP method to interpret the features of the Combined model further enhanced its clinical acceptance.

## Data Availability

The data analyzed in this study is subject to the following licenses/restrictions: The datasets generated or analyzed during the study are available from the corresponding author upon reasonable request. Requests to access these datasets should be directed to Peng-Fei Zhu, 1126277559@qq.com.
