# Supplementary material for: A combined model integrating deep learning, radiomics, and clinical ultrasound features for predicting BRAF V600E mutation in papillary thyroid carcinoma with Hashimoto’s thyroiditis
Source: Front Endocrinol (Lausanne). 2025 Aug 18;16:1641037. doi: 10.3389/fendo.2025.1641037 (PMC12399403; doi:10.3389/fendo.2025.1641037)
Supplement: Supplementary file 1 [file Presentation1.pdf]

Feature Selection Strategy

1. Z-score Normalization

To eliminate dimensional differences between features, Z-score normalization was applied to all input variables using the formula:

$$z=\frac{x-\mu}{\sigma}$$

where μ is the mean and σ is the standard deviation. This step ensures all features are on a comparable scale to facilitate downstream correlation analysis and regularized modeling.

2. Pearson Correlation Filtering

Pearson correlation coefficients were computed between all feature pairs in the normalized training set. For any pair with an absolute correlation greater than 0.9, only the feature with higher information content was retained to reduce redundancy and improve model stability.

3. Minimum Redundancy Maximum Relevance (mRMR)

Following correlation filtering, the mRMR algorithm was used to select features that are highly correlated with the outcome while minimally redundant. A consistent number of 21 features was selected across all models to maintain a balance between feature richness and model complexity.

4. LASSO Regression Compression

LASSO (Least Absolute Shrinkage and Selection Operator) regression was employed to further compress the feature space. This method introduces an L1 regularization term that drives less informative features' coefficients toward zero.

To avoid overfitting, the regularization parameter α was automatically determined through 10-fold cross-validation. Only features with non-zero regression coefficients were retained for model training.

This process was uniformly applied across all modeling pipelines to ensure methodological consistency and improve generalization performance.

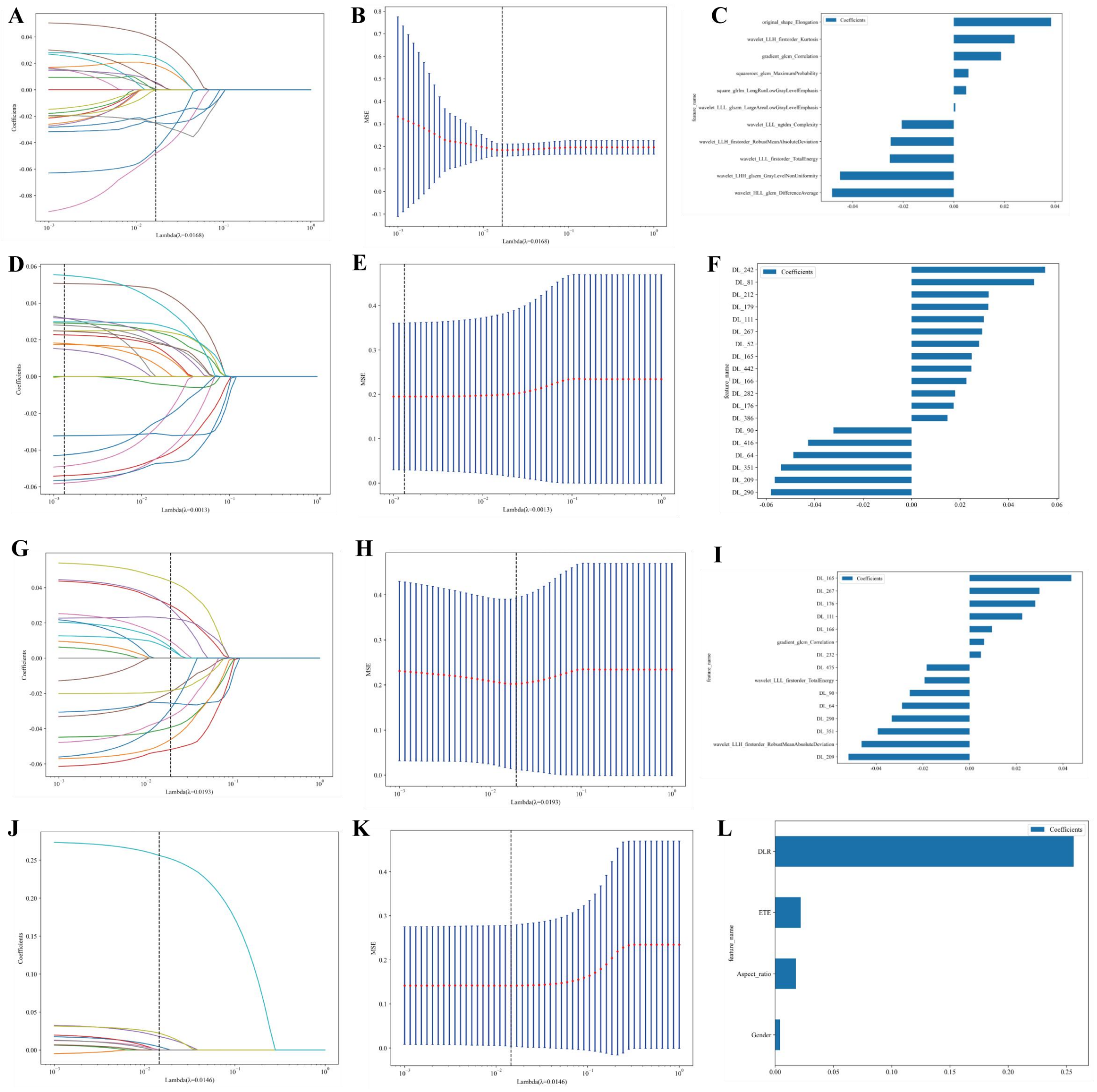

Supplementary Figure 1. LASSO feature selection for the four predictive models.LASSO regression was applied to identify key features for the RAD model (A–C), the DL model (D–F), the DL_RAD model (G–I), and the Combined model (J–L). For each model, the coefficient path (left), the cross-validation curve for optimal λ (middle), and the selected features with non-zero coefficients (right) are presented.

Supplementary Table 1. Number of features retained at each step of feature selection.

**Model**	**Features After Pearson Filtering**	**Features Retained by mRMR**	**Features Selected by LASSO**
Rad Model	151	21	11
DL Model	512	21	19
DL_RAD Model	844	21	15
Combined	10	10	4
